# A novel de novo *RNF216* mutation associated with autosomal recessive Huntington‐like disorder

**DOI:** 10.1002/acn3.51047

**Published:** 2020-05-02

**Authors:** Ke‐Liang Chen, Gui‐Xian Zhao, He Wang, Lei Wei, Yu‐Yuan Huang, Shi‐Dong Chen, Bi‐Ying Lin, Qiang Dong, Mei Cui, Jin‐Tai Yu

**Affiliations:** ^1^ Department of Neurology and Institute of Neurology Huashan Hospital Shanghai Medical College Fudan University Shanghai China; ^2^ Institute of Science and Technology for Brain‐Inspired Intelligence Fudan University Shanghai China

## Abstract

Mutations in *RNF216* have been found to be associated with autosomal recessive Huntington‐like disorder. Here, we describe a patient with Huntington‐like disorder caused by a novel de novo *RNF216* mutation. The patient started to have choreatic movements of both hands, slowly progressing to head, face, and four extremities, with prominent cognitive deterioration. White matter lesions in cerebral hemispheres and brainstem, cerebellar atrophy, and low gonadotropin serum levels have been demonstrated. We have identified a homozygous deletion of exon 2 in the *RNF216* gene by whole‐exome sequencing. Our findings increased genetic knowledge of autosomal recessive Huntington‐like disorder and extended the ethnic distribution of *RNF216* mutations.

## Introduction

Huntington‐like disorder (HDL) was described with characteristic clinical features of chorea, behavioral disturbances, dementia, and lack of the CAG repeat expansion in the *HTT* gene.^1^ In the last few years, a number of different mutations in genes, such as *C9orf72*, *ATXN1*, *ATXN2*, *ATXN3*, *PRNP*, *TBP*, *JPH3*, *DRPLA*, *PANK2*, and *FTL*, have been shown to associate with HDL.^1^, ^2^ Recently, Santens et al.^3^ have reported that *RNF216* mutations were a rare cause of autosomal recessive HDL. Here, we report a Chinese patient with autosomal recessive HDL caused by a novel de novo mutation in the *RNF216* gene.

## Methods

### Subjects

The HDL patient and her family members were referred to the neurological ward at Huashan Hospital affiliated to Fudan University in Shanghai for clinical evaluation and diagnostic investigations. All the subjects, including the proband and her parents, provided written informed consent.

### Clinical evaluation

Medical histories and physical examinations were analyzed. Ancillary tests, such as hormonal evaluation, brain MRI, and neurologic examination, were performed in the proband and her parents. Brain DTI images were acquired and compared with those from three gender‐ and age‐ (<5 years apart) matched healthy controls. The parameters assessed in the patient were defined as abnormal when the mean values were more than 2 standard deviations lower or higher than those of the normal controls.

### Genetic analyses

First, the proband had been previously screened by our targeted sequencing panel (*HTT, C9orf72*, *ATXN1*, *ATXN2*, *ATXN3*, *FRDA*, *PRNP*, *TBP*, *JPH3*, *DRPLA*, *CHAC*, *XK*, *TITK1*, *PANK2*, and *FTL*). Mutations in these genes were excluded*.* Subsequently, whole‐exome sequencing was performed using the Illumina Hiseq sequencing platform. Three exons (exons 2, 3, and 17) of the *RNF216* gene were amplified by real‐time quantitative PCR and analyzed by electrophoresis in her parents.

## Results

The proband was a 38‐year‐old female with normal physical and intellectual development. After graduating from a technical secondary school, she became a kindergarten teacher. She developed choreatic movements of both hands at the age of 29. In the next year, she progressed to choreatic movements of head, face, and four extremities, which incapacitated her for work. Her mother also noted mastatrophy and intermittent lactation, but could not remember more details. The patient was diagnosed with dysmyotonia in another hospital and began treatment with haloperidol, trihexyphenidyl, and tiapride. Symptoms were controlled in short time. Then her disease progressively worsened, and there was a need for support in daily activities to the degree that she became totally dependent. She also developed prominent cognitive deterioration 2 years ago. A comprehensive neuropsychological battery was administrated at the local hospital on 21 November 2018. The intelligence quotient and memory quotient were 57 and 15, respectively, based on Wechsler Adult Intelligence Scale‐Revised China (WAIS‐RC)^4^ and Wechsler Memory Scale.^5^ The Hamilton Anxiety and Depression Rating Scale (HAMA and HAMD) indicated that she displayed mild anxiety and depression (HAMA score = 12 and HAMD score = 14).

**Figure 1:**
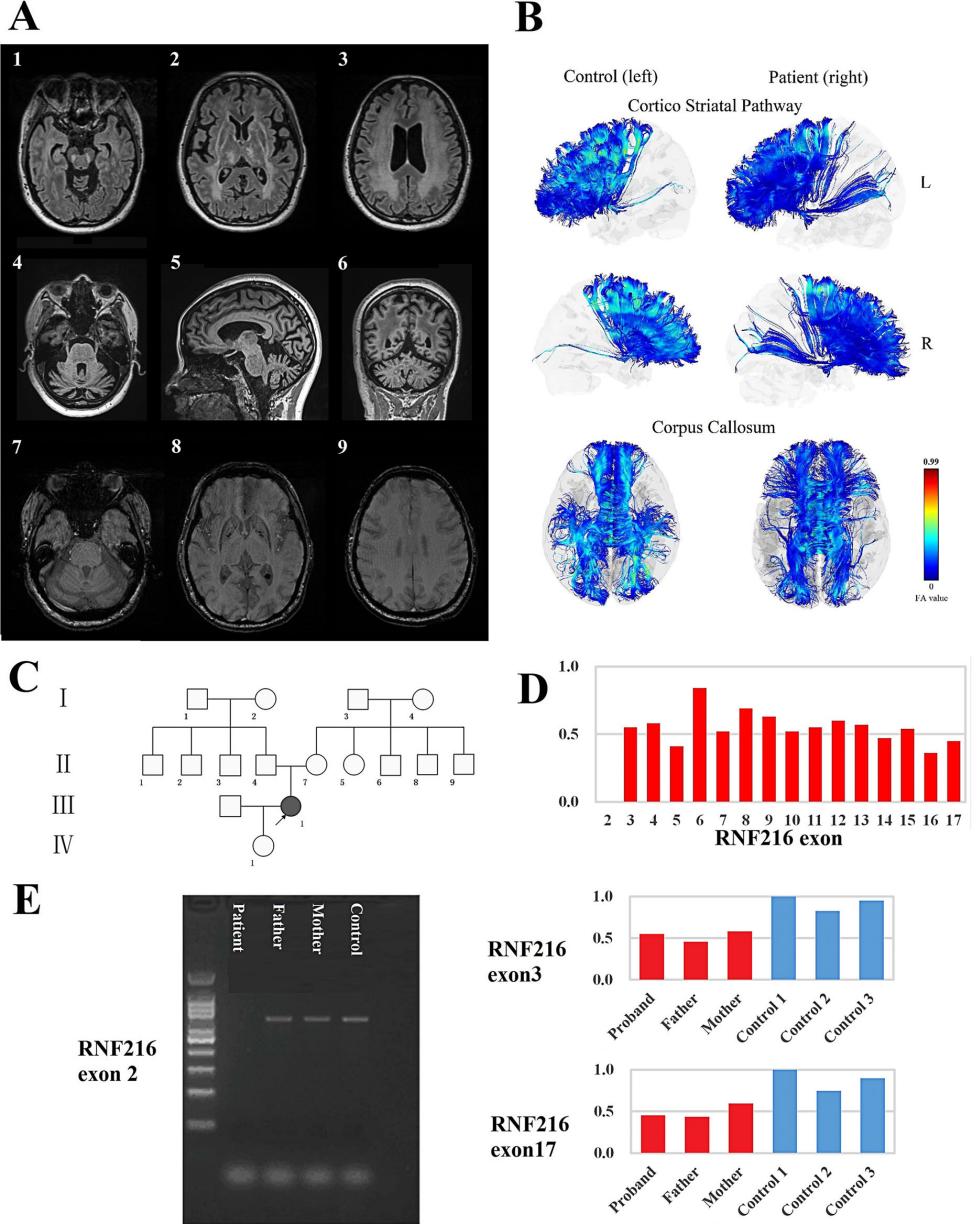
Clinical features of the proband and segregation analysis of the *RNF216* mutations. (A) MRI scan showing extensive bilateral white matter lesions in both cerebral hemispheres and brainstem on T2‐weighted fluid‐attenuated inversion recovery image (1, 2, 3), cerebellar atrophy on T1‐weighted image (4, 5, 6), and no cerebral microbleeds on susceptibility‐weighted imaging (7, 8, 9). (B) Decreased fractional anisotropy (FA) was detected in cortico‐striatal pathway and corpus callosum, when compared with controls. (C) The pedigree of the Huntington‐like disorder family. (D) *RNF216* mutations by whole‐exome sequencing. (E) Segregation analysis of deletions of exons 2, 3, and 17 in the *RNF216* gene by quantitative polymerase chain reaction.

On examination, she looked skinny and body mass index was 18.49 (156 cm in height and 45 kg in weight). The Mini‐Mental State Examination (MMSE) and Montreal Cognitive Assessment (MoCA) scores were 10 and 6, respectively, suggesting that she had dementia. Facial and appendicular choreatic movements were present. Saccadic eye movements were characterized by variable onset latency and speed. Dysarthria was marked. The basal hormonal evaluation revealed inappropriately low luteinizing hormone (<0.1 mUI/mL), as well as low follicular‐stimulating hormone (1.19 mUI/mL) and estradiol (<18.4 pg/mL). Prolactin and cortisol levels were within normal range. Pelvic echography revealed a postmenopausal uterus (33 × 21 × 35 mm) and the ovaries were not seen. Brain MRI was also performed. The results showed moderate cerebellar atrophy on T1‐weighted image, and extensive bilateral white matter lesions in both cerebral hemispheres, as well as in the brainstem on T2‐weighted fluid‐attenuated inversion recovery image. Susceptibility‐weighted imaging showed no cerebral microbleeds in cortical and subcortical areas (Fig. 1A). DTI images displayed widespread white matter deterioration as reflected by the decreased fractional anisotropy values and increased radial diffusivity and axial diffusivity values in the voxels of both hemispheres compared with those of controls, while cerebellar white matter fibers relatively spared (Fig. 1B and Table S1).

She was born through a normal full‐term delivery of nonconsanguinous parents (Fig. 1C). No cognitive impairment was noted in her parents based on their MMSE and MoCA scores (Father: MMSE = 27 and MoCA = 28, Mother: MMSE = 25 and MoCA = 25). Their hormone levels were within normal range. The brain MRI was normal for both of them, consistent with the normal results from a neurologic exam.

We found a homozygous deletion of exon 2 in the *RNF216*, which her parents did not show, determining a novel de novo *RNF216* mutation. No pathogenetic mutations in other HDL‐associated genes were found in the proband. Heterozygous deletions of every exon from 3 to 17 were also found and the parental studies demonstrated that both parents carried heterozygous deletions of exons 3 and 17 (Fig. 1D and 1E).

## Discussion

*RNF216* encodes E3 ubiquitin ligase which recognizes and facilitates the ubiquitination of its target for degradation by the ubiquitin‐proteasome system. *RNF216*‐mediated neurodegeneration has been discovered very recently. Margolin et al.^6^ were the first to identify *RNF216* mutations or a combination of mutations in *RNF216* and *OTUD4* in five Gordon Holmes syndrome (GHS) families of Middle Eastern origin. Four subsequent studies with a total of nine patients reported *RNF216* mutations in Belgian, Indian, Middle Eastern, and Argentinean populations.^3^, ^7^, ^8^, ^9^ Our patient was the first *RNF216* mutation carrier identified in Chinese population, suggesting that *RNF216* mutations are pan‐ethnic.

All previously reported cases with homozygous mutations in *RNF216* were individuals with point mutations from consanguineous families or a common ancestor.^3^, ^6^, ^7^, ^8^, ^9^ This patient showed a novel homozygous deletion of exon 2 in the *RNF216* gene, which has not been previously reported. The parental studies found no corresponding deletion in both parents, indicating that this was a de novo mutation. As per the guidelines of the American College of Medical Genetics and Genomics for sequence variant interpretation,^10^ this variation was interpreted as pathogenic. Several reasons account for this. (1) The variant led to exon deletion, which is possibly a deleterious effect (PVS1); (2) her parents were not affected, indicating it was a de novo mutation (PM6); and (3) it was absent in control databases (gnomAD, 1000 Genomes Project, ClinVar). The patient carried heterozygous deletions of exons 3–17, which were not pathogenic based on the parental studies. It is important to emphasize that the ethnic distribution of *RNF216* mutations might have not been fully described yet due to the small number of cases.

The patient displayed cerebellar atrophy, which is consistent with previous research.^3^ In bilateral cerebral hemispheres and brainstem, high T2 signal was found and no cerebral microhemorrhage foci were found, together suggesting no cerebrovascular pathology. White matter fiber tracts in corpus callosum and surrounding the basal ganglia, which have been reported related to Huntington's disease,^11^ were injured on DTI images. While cerebellar white matter fibers, which were associated with ataxia, relatively spared. This suggests that white matter deterioration was related to her movement disorder.

Evidence of hypogonadotropic hypogonadism also links our patient to GHS, an autosomal recessive neurodegenerative disease with characteristic clinical features of ataxia and hypogonadism.^12^ It should be noted that chorea was also described in GHS patients with *RNF216* mutations, and ataxia was also found in HDL patients with *RNF216* mutations.^3^, ^6^ The GHS diagnosis was also supported by other features, including dementia, cerebellar atrophy, and extensive white matter lesions.

In summary, we reported one patient with autosomal recessive HDL as part of the clinical spectrum of the *RNF216*‐mediated neurodegeneration and extended the ethnic distribution of *RNF216* mutations.

## Author Contributions

JTY, CM, and QD contributed to the conception and design, analysis and interpretation, and drafting and revision of manuscript. KLC and GXZ contributed to the genetic analysis, data interpretation, writing, drafting, and revising the manuscript. SDC, YYH, and BYL contributed to cognitive data acquisition and interpretation. HW and LW contributed to the image collection and interpretation.

## Conflict of Interest

The authors have no financial conflict of interest.

## Patient Consent

Parental/Guardian consent obtained.

## Provenance and Peer Review

Not commissioned; externally peer reviewed.

## Supporting information


**Table S1.** Fractional anisotropy (FA), radial diffusivity (RD), and axial diffusivity (AD) values of the patient and controls. Controls are mean (SD).
