# Supplementary material for: A novel de novo RNF216 mutation associated with autosomal recessive Huntington‐like disorder
Source: Ann Clin Transl Neurol. 2020 May 2;7(5):860–4. doi: 10.1002/acn3.51047 (PMC7261743; doi:10.1002/acn3.51047)
Supplement: Supplementary file 1 — Table S1. Fractional anisotropy (FA), radial diffusivity (RD), and axial diffusivity (AD) values of the patient and controls. Controls are mean (SD). [file ACN3-7-860-s001.docx]

**Supplementary table 1** Fractional anisotropy (FA), radial diffusivity (RD) and axial diffusivity (AD) values of the patient and controls

| White matter fiber tracts | FA | | RD | | AD | |
| --- | --- | --- | --- | --- | --- | --- |
|  | Patient | Controls | Patient | Controls | Patient | Controls |
| left acoustic radiation | 0.155* | 0.247 (0.009) | 0.503* | 0.358 (0.001) | 0.626* | 0.523 (0.010) |
| right acoustic radiation | 0.183 | 0.236 (0.042) | 0.476* | 0.36 (0.026) | 0.610* | 0.517 (0.017) |
| left cortico striatal pathway | 0.142* | 0.228 (0.010) | 0.582* | 0.419 (0.021) | 0.713* | 0.584 (0.019) |
| right cortico striatal pathway | 0.161* | 0.226 (0.008) | 0.589* | 0.416 (0.022) | 0.741* | 0.578 (0.024) |
| left cortico spinal tract | 0.214* | 0.292 (0.033) | 0.427* | 0.343 (0.014) | 0.581* | 0.536 (0.013) |
| right cortico spinal tract | 0.270 | 0.305 (0.03) | 0.409* | 0.339 (0.014) | 0.619* | 0.540 (0.002) |
| left corticothalamic pathway | 0.174* | 0.236 (0.017) | 0.536* | 0.386 (0.016) | 0.690* | 0.548 (0.010) |
| right corticothalamic pathway | 0.188* | 0.233 (0.011) | 0.542* | 0.388 (0.019) | 0.711* | 0.549 (0.018) |
| left frontopontine tract | 0.201* | 0.277 (0.022) | 0.466* | 0.351 (0.020) | 0.626* | 0.533 (0.012) |
| right frontopontine tract | 0.208* | 0.271 (0.019) | 0.483* | 0.351 (0.018) | 0.641* | 0.527 (0.009) |
| left occipitopontine tract | 0.174* | 0.273 (0.010) | 0.483* | 0.361 (0.004) | 0.613* | 0.553 (0.005) |
| left optic radiation | 0.197* | 0.276 (0.004) | 0.524* | 0.387 (0.006) | 0.705* | 0.598 (0.014) |
| right optic radiation | 0.177* | 0.273 (0.039) | 0.569* | 0.409 (0.036) | 0.734* | 0.619 (0.027) |
| left parietopontine tract | 0.221* | 0.289 (0.016) | 0.451* | 0.353 (0.008) | 0.619* | 0.546 (0.004) |
| right parietopontine tract | 0.255* | 0.301 (0.003) | 0.443* | 0.350 (0.008) | 0.640* | 0.554 (0.010) |
| right temporopontine tract | 0.205* | 0.260 (0.000) | 0.422* | 0.347 (0.004) | 0.560* | 0.522 (0.008) |
| left arcuate fasciculus | 0.156* | 0.289 (0.003) | 0.545* | 0.398 (0.011) | 0.688* | 0.611 (0.016) |
| right arcuate fasciculus | 0.180* | 0.285 (0.005) | 0.605* | 0.396 (0.018) | 0.784* | 0.605 (0.031) |
| left cingulum | 0.207 | 0.212 (0.006) | 0.463* | 0.398 (0.011) | 0.625* | 0.546 (0.014) |
| right cingulum | 0.219* | 0.196 (0.011) | 0.465* | 0.404 (0.014) | 0.638* | 0.537 (0.009) |
| left extreme capsule | 0.129* | 0.231 (0.010) | 0.604* | 0.419 (0.012) | 0.728* | 0.592 (0.009) |
| left frontal aslant tract | 0.154* | 0.257 (0.008) | 0.585* | 0.416 (0.019) | 0.730* | 0.607 (0.022) |
| right frontal aslant tract | 0.169* | 0.254 (0.009) | 0.608* | 0.415 (0.019) | 0.771* | 0.602 (0.032) |
| left inferior fronto occipital fasciculus | 0.173* | 0.252 (0.007) | 0.514* | 0.395 (0.019) | 0.667* | 0.578 (0.021) |
| right inferior fronto occipital fasciculus | 0.179* | 0.246 (0.008) | 0.532* | 0.402 (0.020) | 0.691* | 0.585 (0.021) |
| left inferior longitudinal fasciculus | 0.176* | 0.257 (0.017) | 0.490* | 0.407 (0.010) | 0.634* | 0.602 (0.011) |
| right inferior longitudinal fasciculus | 0.187* | 0.249 (0.008) | 0.534* | 0.393 (0.015) | 0.702* | 0.574 (0.017) |
| left middle longitudinal fasciculus | 0.138* | 0.244 (0.019) | 0.572* | 0.426 (0.016) | 0.697* | 0.612 (0.017) |
| right middle longitudinal fasciculus | 0.161* | 0.255 (0.019) | 0.602* | 0.423 (0.007) | 0.753* | 0.618 (0.013) |
| left superior longitudinal fasciculus | 0.158* | 0.262 (0.018) | 0.562* | 0.442 (0.033) | 0.701 | 0.645 (0.038) |
| right superior longitudinal fasciculus | 0.191* | 0.287 (0.017) | 0.611* | 0.414 (0.028) | 0.802* | 0.636 (0.031) |
| left U fiber | 0.169* | 0.239 (0.012) | 0.538* | 0.451 (0.010) | 0.683* | 0.637 (0.012) |
| right U fiber | 0.184* | 0.237 (0.003) | 0.568* | 0.452 (0.014) | 0.734* | 0.637 (0.023) |
| left uncinate fasciculus | 0.121* | 0.154 (0.012) | 0.446 | 0.452 (0.022) | 0.527 | 0.564 (0.020) |
| right uncinate fasciculus | 0.134 | 0.161 (0.016) | 0.522* | 0.438 (0.017) | 0.629* | 0.554 (0.012) |
| left vertical occipital fasciculus | 0.198* | 0.241 (0.01) | 0.484* | 0.444 (0.004) | 0.638 | 0.630 (0.007) |
| right vertical occipital fasciculus | 0.214 | 0.234 (0.013) | 0.512* | 0.447 (0.009) | 0.688* | 0.627 (0.004) |
| corpus callosum | 0.233* | 0.298 (0.011) | 0.500* | 0.380 (0.008) | 0.693* | 0.594 (0.004) |
| left cerebellum | 0.116* | 0.135 (0.007) | 0.469 | 0.482 (0.024) | 0.546 | 0.587 (0.026) |
| right cerebellum | 0.121 | 0.128 (0.004) | 0.497 | 0.476 (0.018) | 0.580 | 0.573 (0.021) |
| left inferior cerebellar peduncle | 0.222 | 0.238 (0.016) | 0.359 | 0.350 (0.014) | 0.496 | 0.496 (0.016) |
| right inferior cerebellar peduncle | 0.215 | 0.213 (0.014) | 0.358* | 0.347 (0.005) | 0.484 | 0.471 (0.01) |
| middle cerebellar peduncle | 0.232 | 0.263 (0.017) | 0.360* | 0.326 (0.007) | 0.497* | 0.480 (0.004) |
| superior cerebellar peduncle | 0.244 | 0.254 (0.007) | 0.329 | 0.317 (0.017) | 0.467 | 0.463 (0.021) |
| vermis | 0.116* | 0.151 (0.007) | 0.471* | 0.422 (0.017) | 0.552 | 0.524 (0.025) |
| left central tegmental tract | 0.194 | 0.200 (0.034) | 0.321 | 0.331 (0.015) | 0.414* | 0.447 (0.006) |
| right central tegmental tract | 0.171* | 0.220 (0.005) | 0.307 | 0.302 (0.011) | 0.389 | 0.415 (0.014) |
| left lateral lemniscus | 0.223 | 0.203 (0.031) | 0.369 | 0.372 (0.014) | 0.504 | 0.504 (0.008) |
| right lateral lemniscus | 0.200 | 0.235 (0.026) | 0.368 | 0.331 (0.033) | 0.488 | 0.471 (0.019) |
| left medial lemniscus | 0.180 | 0.218 (0.039) | 0.301 | 0.324 (0.024) | 0.386* | 0.449 (0.013) |
| right medial lemniscus | 0.227 | 0.235 (0.017) | 0.280 | 0.289 (0.017) | 0.377 | 0.408 (0.018) |
| left medial longitudinal fasciculus | 0.163* | 0.192 (0.014) | 0.322 | 0.325 (0.014) | 0.401* | 0.434 (0.015) |
| right medial longitudinal fasciculus | 0.164 | 0.200 (0.024) | 0.319 | 0.317 (0.022) | 0.399 | 0.425 (0.014) |
| left rubrospinal tract | 0.176* | 0.214 (0.005) | 0.309 | 0.313 (0.018) | 0.398 | 0.429 (0.022) |
| right rubrospinal tract | 0.198* | 0.230 (0.008) | 0.312 | 0.312 (0.005) | 0.417 | 0.437 (0.011) |
| left spinothalamic tract | 0.182* | 0.204 (0.009) | 0.328 | 0.334 (0.004) | 0.424* | 0.455 (0.007) |
| right spinothalamic tract | 0.206* | 0.222 (0.007) | 0.313 | 0.3 (0.009) | 0.417 | 0.413 (0.013) |

Note: * indicates abnormal FA, RD or AD values. The parameters assessed in the patient were defined as abnormal when the mean values were more than 2 standard deviations (SDs) lower or higher than those of the normal controls.

Controls are mean (SD).
